# Adaptive group sequential survival comparisons based on log-rank and pointwise test statistics

**DOI:** 10.1177/09622802211043262

**Published:** 2021-10-12

**Authors:** Jannik Feld, Andreas Faldum, Rene Schmidt

**Affiliations:** 352489 Institute of Biostatistics and Clinical Research, 9185 University of Münster, Muenster, Germany

**Keywords:** Adaptive design, phase II trial, phase III trial, sample size recalculation, survival analysis, log–rank, Nelson–Aalen, bivariate, seamless design

## Abstract

Whereas the theory of confirmatory adaptive designs is well understood for uncensored data, implementation of adaptive designs in the context of survival trials remains challenging. Commonly used adaptive survival tests are based on the independent increments structure of the log-rank statistic. This implies some relevant limitations: On the one hand, essentially only the interim log-rank statistic may be used for design modifications (such as data-dependent sample size recalculation). Furthermore, the treatment arm allocation ratio in these classical methods is assumed to be constant throughout the trial period. Here, we propose an extension of the independent increments approach to adaptive survival tests that addresses some of these limitations. We present a confirmatory adaptive two-sample log-rank test that allows rejection regions and sample size recalculation rules to be based not only on the interim log-rank statistic, but also on point-wise survival rate estimates, simultaneously. In addition, the possibility is opened to adapt the treatment arm allocation ratio after each interim analysis in a data-dependent way. The ability to include point-wise survival rate estimators in the rejection region of a test for comparing survival curves might be attractive, e.g., for seamless phase II/III designs. Data-dependent adaptation of the allocation ratio could be helpful in multi-arm trials in order to successively steer recruitment into the study arms with the greatest chances of success. The methodology is motivated by the LOGGIC Europe Trial from pediatric oncology. Distributional properties are derived using martingale techniques in the large sample limit. Small sample properties are studied by simulation.

## Introduction

The log-rank test^1^ is presently the gold standard method for analysing differences in survival data in randomised clinical trials. For this reason adaptive survival tests are commonly based upon the log-rank test statistic and its independent increments structure.^2,3^ However, these designs suffer from some limitations we want to address. One limitation is that effectively only the interim log-rank statistic may be used for design modifications (such as data-dependent sample size recalculation).^4^ Moreover, the treatment arm allocation ratio in these classical methods is assumed to be constant throughout the whole trial period. However, in the context of seamless phase II/III designs or early phase trials it may be desirable to include point-wise survival rates (e.g. 1 year survival rates) in the decision making, since survival rates at a given time-point of interest are chosen as a primary endpoint regularly in such trials. Likewise, data-dependent adaptations of the treatment arm allocation ratio could be helpful in multi-arm trials in order to successively steer recruitment into the study arms with the greatest chances of success. Therefore we propose an extension of the independent increments approach to adaptive survival tests, which can rely on both: (i) the pointwise Neelson Aalen type survival rates estimator and (ii) the log rank test statistic. More specifically our approach extends the commonly used methodology by Wassmer,^3^ which neither supports the use of point-wise survival rate estimates nor foresees data-dependent adaptations of the treatment arm allocation ratio. In doing so, our approach avoids those difficulties associated with alternative methods based on the patient-wise separation principle, which have the common disadvantage that the test procedure may either neglect part of the observed survival data or tend to be conservative. We will show by simulation that our extended methodology maintains the performance of the current standard methodology while offering various new design possibilities.

The methodology presented here is motivated by the LOGGIC Europe trial (Eudra-CT: 2018-000636-10). LOGGIC Europe is a randomized, international multicentre phase III therapy optimization trial for children and adolescents with low–grade glioma. Primary endpoints of the trial are the progression-free survival (PFS) and the disease control rate (DCR). PFS addresses long–term efficacy of treatment and is defined as time from randomization up to progression of disease or death for all reasons whatever occurs first. DCR addresses short–term efficacy of treatment redand is essentially defined as the PFS-rate at some early timepoint.

The paper is organized as follows. We start by settling notation and stochastic assumptions. Section ‘Joint martingale representation of the log–rank statistic and cumulative hazard difference’ presents briefly the bivariate representation of the two test statistics and its distributional properties. The design algorithm and corresponding planning methodology is presented in section ‘Adaptive log–rank test with simultaneous use of interim log–rank statistic and cumulative hazard rate difference’. In section ‘Example: A two–step log–rank test with futility criterion based on short–term survival rate’ we present some example use-case in order to illustrate practical implementation of our method. Small sample properties are studied by simulation in section ‘Simulation’. We conclude with a discussion of our findings and prospects for future research. Mathematical proofs are shifted to the supplemental material.

## Notation and stochastic assumptions

Let (Ω,F,P) denote the probability space upon which all random variables are defined. Unless otherwise specified, random variables are denoted by capital Latin letters, whereas realizations of random variables are denoted by the corresponding lower case Latin letters. We set 0/0:=0 whenever formal division of zero by zero occurs in sequel.

We consider the problem of testing the equality of survival distributions for two treatments A and B , say, based on accumulating survival data across several stages of a sequential design. After each stage a confirmatory (interim) analysis is performed with the possibility for interim decisions (e.g. binding futility stop or sample size recalculation) based on (i) the observed interim log–rank statistic and (ii) interim estimates of $s_{0}$ -years survival rate differences for some prefixed time-point $s_{0}>0$ .

**Figure 1.**
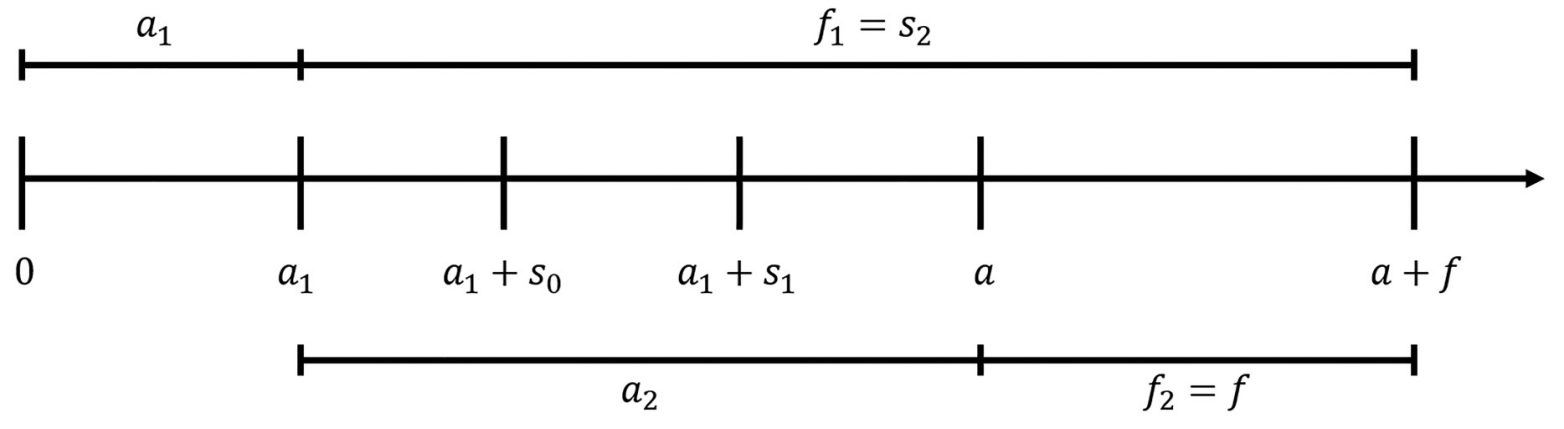
Initial time schedule. At time of the final analysis, first stage patients would have a minimum follow-up of $f_{1}=a_{2}+f>s_{1}$ years under the initial time schedule. Second stage patients would have a minimum follow-up of $f_{2}=f$ at time of the final analysis.

In this context we will assume an initial trial design with l stages. The stages will recruit patients successively, i.e. patients from stage k are recruited between calendar times $\sum_{i=1}^{k-1}a_{i}$ and $\sum_{i=1}^{k}a_{i}$ where $a_{i}>0$ are the recruitment period lengths of the stages. We set $a\;:\;=\sum_{i=1}^{l}a_{i}$ as the overall recruitment period length. The final analysis will be performed at calender time a+f . Patients from stage k will therefore have at least a follow-up period length of $f_{k}=\sum_{i=k+1}^{l}a_{i}+f$ . An example timeline for l=2 stages is given in Figure 1. The planned annual recruitment rate is denoted with r .

For this purpose, let $N_{x,k}$ denote the set of patients from treatment group x=A,B , who entered the trial at stage k (i.e. between calendar time $\sum_{i=1}^{k-1}a_{i}$ and $\sum_{i=1}^{k}a_{i}$ ), and let $n_{x,k}\;:\;=\#N_{x,k}$ denote the number of such patients. Let $N_{k}\;:\;=\underset{x}{⋃}N_{x,k}$ denote the set of all patients from stage k pooled over both treatment groups, and $N\;:\;=\underset{x,k}{⋃}N_{x,k}$ the overall set of trial patients. Let $n_{k}\;:\;=\sum_{x}n_{x,k}$ and $n\;:\;=\sum_{x,k}n_{x,k}$ . The parameter n will index the arrival process and asymptotic results will be derived in the limit n→∞ . Accordingly, we assume that group sizes grow uniformly as total sample size increases, i.e. we assume there exist constants $v_{k}>0$ such that $\#N_{A,k}/n_{k}\to \frac{1}{1+v_{k}}$ and $\#N_{B,k}/n\to \frac{v_{k}}{1+v_{k}}$ in probability as n→∞ . Thus the constants $v_{k}$ are the asymptotic, stagewise allocation ratio between the treatment groups. We furthermore assume that the stages also grow uniformly as total sample size increases, i.e. $\#N_{k}/n=n_{k}/n\to a_{k}/a$ in probability as n→∞ .

To patient i is associated a random triplet $\left\{E_{i},C_{i},T_{i}\right\}$ . $E_{i}$ is the entry time into the study, the possibly infinite random variable $C_{i}$ is the time of censoring after entry, and $T_{i}$ is the survival time after entry. Our stochastic assumptions are as follows: (1) $T_{i}$ , $C_{i}$ and $E_{i}$ are mutually independent for fixed i , and (2) data from different patients are independent and identical distributed within treatment groups.

Based on the observed data, we will calculate the *number of events* in stage k from treatment group x=A,B up to study time s≥0 as

(1) $$D_{x,k}(s)\;:\;=\sum_{i\in N_{x,k}}D_{i}(s),\;\;D_{i}(s)\;:\;=I(T_{i}\leq s,T_{i}\leq C_{i}),$$

and the *number at risk* by study time s≥0 in stage k and treatment group x=A,B as

(2) $$Y_{x,k}(s)\;:\;=\sum_{i\in N_{x,k}}I(T_{i}\wedge C_{i}\geq s).$$

Finally, let $J_{x,k}(s)\;:\;=I(Y_{x,k}(s)>0)$ and $L_{k}(s)$ the log-rank weight factor

(3) $$L_{k}(s)\;:\;=\frac{Y_{A,k}(s)\cdot Y_{B,k}(s)}{Y_{A,k}(s)+Y_{B,k}(s)}.$$

For each s≥0 , let $F_{s}$ be the σ –algebra generated by

(4) $$\begin{array}{rl} & I\{T_{i}\leq s\wedge C_{i}\},\;T_{i}\cdot I\{T_{i}\leq s\wedge C_{i}\},\\ & I\{C_{i}\leq s\wedge T_{i}\},\;C_{i}\cdot I\{C_{i}\leq s\wedge T_{i}\}, \end{array}$$

for i∈N . We consider $D_{x,k}$ , $Y_{x,k}$ , $J_{x,k}$ , $L_{k}$ as stochastic process in study time s≥0 , adapted to the filtration $(F_{s}{)}_{s\geq 0}$ . The filtration $(F_{s}{)}_{s\geq 0}$ comprises the information that is observed in the study. Whenever we want to emphasize the dependence of above processes on n∈N , we will index them additionally by n e.g. $D_{x,k}^{n}$ instead of $D_{x,k}$ .

As usual, we let $\lambda_{x}(s)\;:\;=\lim_{\delta \to 0}P(s\leq T_{i}<s+\delta |T_{i}\geq s)/\delta$ denote the hazard of a patient i from treatment group x=A,B . We denote by $\Lambda_{x}(s)\;:\;=\int_{0}^{s}\lambda_{x}(u)du$ and $S_{x}(s)\;:\;=\exp\;(-\Lambda_{x}(s))\equiv P(T_{i}>s)$ the corresponding cumulative hazard and survival functions for treatment group x=A,B , respectively.

In this context, we consider testing the two–sided null hypothesis

(5) $$H_{0}\;:\;S_{A}(s)=S_{B}(s)\;\mathrm{for}\;\mathrm{all}\;0\leq s\leq s_{\max}$$

that the survival functions in the two treatment arms coincide within some prefixed interval $\left[0,s_{\max}\right]$ .

We proceed as follows to test $H_{0}$ . Using martingale techniques, we will first derive the joint distribution of (i) the stage–wise log–rank test statistics and (ii) the stage–wise difference in the Nelsen–Aalen estimates between the two treatment arms evaluated at some prefixed study time $s_{0}$ . On this basis, we provide a confirmatory adaptive two–sample log–rank test where provision is made for interim decision making and design modifications based on both (i) the interim log–rank statistic and (ii) interim estimates of the cumulative hazard rate differences at timepoint $s_{0}$ . With a view to practical application, sample size recalculation is one of the most common design modifications. Therefore, sample size recalculation based on conditional power will be elaborated and studied in detail, analytically and by simulation.

## Joint martingale representation of the log–rank statistic and cumulative hazard difference

The weighted two–sample log–rank statistic in stage k is defined as

(6) $$\begin{array}{rl} LR_{k}(s)\;: & =\sum_{\begin{array}{c} i\in N_{A,k}\\ D_{i}(s)=1 \end{array}}\frac{L_{k}(T_{i})}{Y_{A,k}(T_{i})}-\sum_{\begin{array}{c} i\in N_{B,k}\\ D_{i}(s)=1 \end{array}}\frac{L_{k}(T_{i})}{Y_{B,k}(T_{i})}, \end{array}$$

where $L_{k}$ is the weight from equation (3). The difference of the group–wise Nelson–Aalen estimates in stage k is given as

(7) $$\begin{array}{rl} \Delta_{k}(s)\;: & =\sum_{\begin{array}{c} i\in N_{A,k}\\ D_{i}(s)=1 \end{array}}\frac{J_{A,k}(T_{i})}{Y_{A,k}(T_{i})}-\sum_{\begin{array}{c} i\in N_{B,k}\\ D_{i}(s)=1 \end{array}}\frac{J_{B,k}(T_{i})}{Y_{B,k}(T_{i})}, \end{array}$$

which are both $F_{s}$ –adapted processes. It follows from theorem A2, that under mild regularity assumptions and the proportional hazards assumption $\lambda_{B}=\omega \lambda_{A}$ for some ω>0 , the following distributional approximation holds:

(8) $$\left(\begin{array}{c} \frac{n_{k}^{-1/2}LR_{k}(s)}{\sqrt{\left[M_{k}^{LR}](s\right)}}\\ \frac{n_{k}^{1/2}\Delta_{k}(s)}{\sqrt{\left[M_{k}^{\Delta }](s\right)}} \end{array}\right)\overset{D}{\approx }\;N(\left(\begin{array}{c} -\sqrt{n_{k}}\log\;(\omega )\cdot \sigma_{LR,k}(s)\\ -\sqrt{n_{k}}\log\;(\omega )\cdot \frac{\Lambda_{A}(s)}{\sigma_{\Delta ,k}(s)} \end{array}\right),\left(\begin{array}{cc} 1 & \frac{\Lambda_{A}(s)}{\sigma_{LR,k}(s)\sigma_{\Delta ,k}(s)}\\ \frac{\Lambda_{A}(s)}{\sigma_{LR,k}(s)\sigma_{\Delta ,k}(s)} & 1 \end{array}\right)),$$

where $\sigma_{LR,k}^{2}(s)\;:\;=plim_{n_{k}\to \infty }D_{k}(s)/n_{k}\cdot \frac{v_{k}}{(1-v_{k}{)}^{2}}$ and $\sigma_{\Delta ,k}^{2}(s)$ are some deterministic functions (see equations (14) and (13) below) and

(9) $$\begin{array}{rl} \left[M_{k}^{LR}](s\right) & \;:\;=n_{k}^{-1}\sum_{\begin{array}{c} i\in N_{A,k}\\ D_{i}(s)=1 \end{array}}\frac{L_{k}(T_{i}{)}^{2}}{Y_{A,k}(T_{i}{)}^{2}}+n_{k}^{-1}\sum_{\begin{array}{c} i\in N_{B,k}\\ D_{i}(s)=1 \end{array}}\frac{L_{k}(T_{i}{)}^{2}}{Y_{B,k}(T_{i}{)}^{2}},\\ \left[M_{k}^{\Delta }](s\right) & \;:\;=n_{k}\sum_{\begin{array}{c} i\in N_{A,k}\\ D_{i}(s)=1 \end{array}}\frac{J_{A,k}(T_{i})}{Y_{A,k}(T_{i}{)}^{2}}+n_{k}\sum_{\begin{array}{c} i\in N_{B,k}\\ D_{i}(s)=1 \end{array}}\frac{J_{B,k}(T_{i})}{Y_{B,k}(T_{i}{)}^{2}}. \end{array}$$

The left hand side of (8) has also approximately independent, bivariate normal distributed increments as stated in theorem A2.

For given ω>0 we set

(10) $$\mu_{k}\;:\;=-\sqrt{n_{k}}\log\;(\omega ).$$

In practice the time-dependant correlation parameter on the right hand side of (8) is unknown. However, for a fixed time point $s_{0}>0$ it can be consistently estimated at time of the interim analysis (see (24)). Under further planing assumptions it is possible to deduce closed formulas for the functions $\sigma_{LR,k}$ and $\sigma_{\Delta ,k}$ . Assuming (in addition to above mentioned mild regularity conditions of theorem A2):

∙ No loss to follow-up:

(11) $$\forall i\in N_{k}\;:\;E_{i}+C_{i}\equiv a+f$$

∙ Uniform recruitment:

(12) $$\forall i\in N_{k}\;:\;E_{i}∼U([\sum_{i=1}^{k-1}a_{i},\sum_{i=1}^{k}a_{i}])$$

the following two equations hold (see appendix for proofs):

(13) $$\sigma_{\Delta ,k}^{2}(s)=\left\{ \begin{array}{cc} \frac{(1+v_{k}{)}^{2}}{v_{k}}\left(\frac{1}{S_{A}(s)}-1\right), & \mathrm{if}\;s\leq f_{k}\\ \frac{(1+v_{k}{)}^{2}}{v_{k}}\left(\frac{1}{S_{A}(f_{k})}-1+\int_{\;f_{k}}^{s}\frac{\lambda_{A}(u)\cdot a_{k}}{S_{A}(u)\cdot (a_{k}+f_{k}-u)}du\right), & \mathrm{if}\;f_{k}<s<a_{k}+f_{k} \end{array}\right.$$

(14) $$\sigma_{LR,k}^{2}(s)=\left\{ \begin{array}{cc} \frac{v_{k}}{(1+v_{k}{)}^{2}}(1-S_{A}(s)), & \mathrm{if}\;s\leq f_{k}\\ \frac{v_{k}}{(1+v_{k}{)}^{2}}\left(1-\frac{a_{k}+f_{k}-s}{a_{k}}S_{A}(s)-\frac{1}{a_{k}}\int_{\;f_{k}}^{s}S_{A}(u)du\right), & \mathrm{if}\;f_{k}<s<a_{k}+f_{k}\\ \frac{v_{k}}{(1+v_{k}{)}^{2}}\left(1-\frac{1}{a_{k}}\int_{\;f_{k}}^{a_{k}+f_{k}}S_{A}(u)du\right), & \mathrm{if}\;s\geq a_{k}+f_{k} \end{array}\right.$$

## Adaptive log–rank test with simultaneous use of interim log–rank statistic and cumulative hazard rate difference

### The design algorithm

For the sake of notational simplicity we will focus on two-step designs in the sequel (i.e. l=2 ). The two–step adaptive design will proceed as follows: Assume an initial design with accrual of patients between calender time 0 and a years, and a final analysis at calender time a+f (corresponding to minimum follow–up period of f years). We assume that the value of f is prefixed by clinical consideration. Choice of a will be detailed in section ‘Initial sample size calculation’ based on power arguments. Patients recruited prior to calender time $a>a_{1}>0$ define the set of first stage patients $N_{1}$ , and patients recruited between calendar time $a_{1}$ and $a\;:\;=a_{1}+a_{2}$ define the set of second stage patients $N_{2}$ . The interim analysis will take place at time $a_{1}+s_{1}$ for some $0<s_{1}<a_{2}$ and will include the patients of stage one with their first $s_{1}$ years of follow-up.

At the interim analysis the log–rank statistic in stage 1 patients based on information up to study time $s_{1}$

(15) $$Z_{1}^{*}\;:\;=Z_{11}\;:\;=n_{1}^{-1/2}\cdot \frac{LR_{1}(s_{1})}{\sqrt{\left[M_{1}^{LR}](s_{1}\right)}}$$

and the standardized cumulative hazard rate difference at some prefixed (early) study time $0<s_{0}\leq s_{1}$

(16) $$B_{1}\;:\;=n_{1}^{1/2}\cdot \frac{\Delta_{1}(s_{0})}{\sqrt{\left[M_{1}^{\Delta }](s_{0}\right)}}$$

will be calculated. $B_{1}$ is an interim estimate of the difference in short–term response. More specifically, $\Delta_{1}(s_{0})$ is an interim estimate of $\log\;(S_{B}(s_{0})/S_{A}(s_{0}))$ . The design algorithm is as follows: The design stops at the interim analysis with rejection of $H_{0}$ if the observed value $z_{1}$ for $Z_{1}^{*}$ exceeds some critical value $u_{1}$ . The design stops for futility if either $z_{1}$ falls below some futility bound $u_{0}$ or if the observed value $b_{1}$ for $B_{1}$ drops below some prefixed boundary $b_{0}$ . Otherwise, if $u_{0}\leq z_{1}<u_{1}$ and $B_{1}>b_{0}$ , the design continues to stage two. At this time, the recruitment period length of stage two $a_{2}$ can be data–dependent recalculated. The recalculated recruitment period length $a_{2}^{\prime }\;:\;=a_{2}^{\prime }(Z_{1}^{*},B_{1})$ of stage two is chosen in dependence of the observed values for $Z_{1}^{*}$ and $B_{1}$ subject to the constraint $s_{1}<a_{2}^{\prime }\leq a_{\max}-a_{1}$ . Here, $a_{\max}>a_{1}$ denotes a maximum trial recruitment period length that is fixed in advance in order to avoid unrealistic or unfeasible trial duration. We set $a^{\prime }\;:\;=a_{1}+a_{2}^{\prime }$ and $f_{1}^{\prime }\;:\;=a_{2}^{\prime }+f$ .

The final analysis will take place at calendar time $a^{\prime }+f$ and will include both, patients of stage one $N_{1}$ with their full follow-up data of at least $f_{1}^{\prime }$ years and the set of second stage patients $N_{2}$ with their follow-up time of at least f years. At the time of the final analysis, the increment of the log–rank statistic in stage one patients beyond study time $s_{1}$ will be calculated

(17) $$Z_{12}\;:\;=n_{1}^{-1/2}\cdot \frac{LR_{1}(a^{\prime }+f)-LR_{1}(s_{1})}{\sqrt{\left[M_{1}^{LR}](a^{\prime }+f)-[M_{1}^{LR}](s_{1}\right)}},$$

as well as the log–rank statistic of stage two patients

(18) $$Z_{22}\;:\;=n_{2}^{-1/2}\cdot \frac{LR_{2}(f_{1}^{\prime })}{\sqrt{\left[M_{2}^{LR}](f_{1}^{\prime }\right)}}.$$

Notice that $Z_{12}$ and $Z_{22}$ are conditionally independent given $Z_{1}^{*}$ and $B_{1}$ .

The null hypothesis $H_{0}$ will be rejected at the final analysis if the second stage test statistic

(19) $$Z_{2}^{*}\;:\;=\frac{\sqrt{\eta_{11}}\cdot Z_{11}+\sqrt{\eta_{12}-\eta_{11}}\cdot Z_{12}+\sqrt{\eta_{22}}\cdot Z_{22}}{\sqrt{\eta_{12}+\eta_{22}}}$$

exceeds some critical value $u_{2}$ , where the prefixed weight factors

(20) $$\eta_{11}=\sigma_{LR,1}^{2}(s_{1}),\;\eta_{12}=\sigma_{LR,1}^{2}(a_{1}+f_{1}),\;\eta_{22}=\sigma_{LR,2}^{2}(f_{1}),$$

amount to the expected variance of the log–rank statistics under some initial planning alternative $K_{1}$ (see section ‘Calculation of the critical bounds’). Their values are given in (13) and (14). The weights $\eta_{ij}$ have to be fixed in advance and remain unchanged while the trial is ongoing.

### The rejection region

The design algorithm described in section 4.1 corresponds to the rejection region

(21) $$R\;:\;=\{Z_{1}^{*}\geq u_{1}\}\cup \{B_{1}>b_{0},u_{0}\leq Z_{1}^{*}<u_{1},Z_{2}^{*}\geq u_{2}\}$$

of the null hypothesis $H_{0}$ . It is crucial that the design parameters $b_{0}$ , $\eta_{11}$ , $\eta_{12}$ , $\eta_{22}$ and $0<s_{0}\leq s_{1}<f$ as well as the critical bounds $u_{0}$ , $u_{1}$ are prefixed and remain unchanged during the trial. Note that the critical bound $u_{2}$ will be calculated at the interim analysis according to formula 22 when the correlation ρ of $Z_{1}$ and $B_{1}$ can be estimated to obtain a rejection region which exhausts the full significance level. The calculation of the critical bounds $b_{0}$ , $u_{0}$ , $u_{1}$ , $u_{2}$ is elaborated next.

### Calculation of the critical bounds

The rejection region R defines a level α test of the null hypothesis $H_{0}$ if the critical bounds $b_{0}$ , $u_{0}$ , $u_{1}$ , $u_{2}$ are chosen according to the proviso that $P_{H_{0}}(R)=\alpha$ , i.e. such that

(22) $$\alpha =\Phi (-u_{1})+\int_{u_{0}}^{u_{1}}\Phi \left(\frac{\sqrt{\eta_{11}}z_{1}-\sqrt{\eta_{12}+\eta_{22}}u_{2}}{\sqrt{\eta_{12}-\eta_{11}+\eta_{22}}}\right)\Phi \left(\frac{\rho z_{1}-b_{0}}{\sqrt{1-\rho^{2}}}\right)\phi (z_{1})dz_{1}.$$

Notice that the critical bounds depend on the nuisance parameter

(23) $$\rho =\frac{\Lambda_{A}(s_{0})}{\sigma_{\Delta ,1}(s_{0})\sigma_{LR,1}(s_{1})},$$

which is in fact unknown during the trial if one does not know the true hazard function $\lambda_{A}$ . However, it may be estimated consistently at time of the interim analysis via

(24) $$\hat{\rho }\;:\;=\frac{\left[M_{1}^{LR},M_{1}^{\Delta }](s_{0}\right)}{\sqrt{\left[M_{1}^{\Delta }](s_{0})\cdot [M_{1}^{LR}](s_{1}\right)}}.$$

Nevertheless there are infinite parameter combinations of the critical bounds which satisfy equation (22). It is therefore crucial, that one parameter constellation $\left(u_{0},b_{0},u_{1}\right)$ is chosen in advance and remains unchanged during the trial. The critical bound $u_{2}$ will then be calculated at the interim analysis as the unique solution to (22) with $\hat{\rho }$ plugged in for ρ .

### Initial sample size calculation

Initial sample size calculation is performed under the planning alternative hypothesis

(25) $$K_{1}\;:\;S_{B}=S_{A}^{\omega_{0}}$$

and under the assumption, that no sample size recalculation is performed i.e. $a_{2}^{\prime }=a_{2}$ . For the initial sample size calculation we need to fix the proportion $\pi \;:\;=a_{1}/a\in (0,1)$ of accrual to stage 1. Note that the weights $\eta_{ij}$ are fixed in advance and must not be changed while the trial is ongoing. In fact they have to be calculated simultaneously with the sample size. For given weight factors $\eta_{ij}$ , the condition to reject null hypothesis $H_{0}$ with probability 1−β under planning alternative $K_{1}$ is $P_{K_{1}}(R)=1-\beta$ . Using the distribution approximation (8) this proviso is tantamount to

(26) $$1-\beta =P_{K_{1}}(Z_{1}^{*}\geq u_{1})+P_{K_{1}}(B_{1}>b_{0},u_{0}\leq Z_{1}^{*}<u_{1},Z_{2}^{*}\geq u_{2}).$$

Notice that $B_{1}$ and $Z_{2}^{*}$ are independent given $Z_{1}^{*}$ . Thus the right hand side of (26) equals

(27) $$\Phi (\mu_{1}\sigma_{LR,1}(s_{1})-u_{1})+\int_{u_{0}}^{u_{1}}\phi (z_{1}-\mu_{1}\sigma_{LR,1}(s_{1}))\cdot P_{K_{1}}(B_{1}>b_{0}|Z_{1}^{*}=z_{1})\cdot P_{K_{1}}(Z_{2}^{*}\geq u_{2}|Z_{1}^{*}=z_{1})dz_{1}.$$

Using again the distribution approximation (8) we get the identities

(28) $$P_{K_{1}}(B_{1}>b_{0}|Z_{1}^{*}=z_{1})=\Phi \left(-\frac{b_{0}-\mu_{k}\frac{\Lambda_{A}(s_{0})}{\sigma_{\Delta ,1}(s_{0})}-\rho (z_{1}-\mu_{1}\sigma_{LR,1}(s_{1}))}{\sqrt{1-\rho^{2}}}\right)$$

and

(29) $$\begin{array}{rl} & P_{K_{1}}(Z_{2}^{*}\geq u_{2}|Z_{1}^{*}=z_{1})\\ & =\Phi \left(-\frac{u_{2}\cdot \sqrt{\eta_{12}+\eta_{22}}-\sqrt{\eta_{11}}z_{1}-\sqrt{\eta_{12}-\eta_{11}}\cdot (\mu_{1}\sqrt{\sigma_{LR,1}^{2}(a_{1}+f_{1})-\sigma_{LR,1}^{2}(s_{1})})-\sqrt{\eta_{22}}\mu_{2}\sigma_{LR,2}(f_{1})}{\sqrt{\eta_{12}-\eta_{11}+\eta_{22}}}\right) \end{array}$$

Using the identities (28) and (29), formulas (13) and (14) for $\sigma_{LR,k}(s)$ and $\sigma_{\Delta ,k}(s)$ and the identity $n_{k}=r\cdot a_{k}$ in equation (26), one can solve (26) and (22) numerically to obtain the critical bound $u_{2}$ and the needed recruitment period lengths $a_{1}$ and $a_{2}$ . We provide R syntax in the supplemental material to do so.

At the interim analysis, $a_{2}$ and thus $n_{2}$ may be modified in a data–dependent way to keep up adequate power performance of the trial, as will be detailed in the next section.

### Data–dependent sample size recalculation at the interim analysis based on conditional power

At the interim analysis, we are free to revise the length of the stage two accrual period $a_{2}$ in the light of $Z_{1}^{*}$ (interim log–rank statistic) and $B_{1}$ (observed difference in short–term response) without compromising type I error rate control. This is a consequence of the independent increments structure of the bivariate process given by the left hand side of (8). For this purpose, we will first calculate the required length of the accrual period $a_{2}^{CP}$ to achieve a desired conditional power. To avoid unrealistic large trial duration, the revised length of the accrual period will finally be chosen as

(30) $$a_{2}^{\prime }\;:\;=\max(\min(a_{2}^{CP},a_{\max}-a_{1}),s_{1}).$$

Recall that $a_{1}+s_{1}$ is the calendar time of the interim analysis and $a_{\max}$ is a prefixed maximum trial recruitment period length.

Likewise, we are free to revise the allocation ratio between treatment groups in the light of $Z_{1}^{*}$ and $B_{1}$ . Let $v_{2}^{\prime }$ denote the revised allocation ratio of stage two patients to treatment group B as referred to treatment group A. Furthermore we may use an updated recruitment rate $r^{\prime }$ to adjust for new experience.

To calculate $a_{2}^{CP}$ , we estimate the true hazard ratio ω via

(31) $$\hat{\omega }\;:\;=\exp\;\left(-\frac{1}{n}\frac{LR_{1}(s_{1})}{\left[M_{1}^{LR}](s_{1}\right)}\right).$$

Notice that $LR_{1}(s_{1})$ and $N_{1}(s_{1})$ are observed at the interim analysis. We can also estimate $\sigma_{LR,1}(s_{1})$ consistently at the interim analysis through the estimator ${\hat{\sigma }}_{LR,1}^{2}(s_{1})\;:\;=[M_{1}^{LR}](s_{1})$ . Sample size recalculation will be performed under the revised planning alternative

(32) $$K_{1}^{\prime }\;:\;\;S_{B}=S_{A}^{\hat{\omega }}$$

suggested by the observed interim estimate $\hat{\omega }$ of the true hazard ratio. The condition to achieve a conditional power $P_{K_{1}^{\prime }}(Z_{2}^{*}\geq u_{2}|Z_{1}^{*}=z_{1},B_{1}=b_{1})$ of $1-\beta_{2}$ under the revised planning alternative $K_{1}^{\prime }$ is equivalent to

(33) $$\begin{array}{rl} & \Phi^{-1}(\beta_{2})=\\ & \frac{u_{2}\sqrt{\eta_{12}+\eta_{22}}-\sqrt{\eta_{11}}z_{1}-\sqrt{\eta_{12}-\eta_{11}}\cdot {\hat{\mu }}_{1}\sqrt{\sigma_{LR,1}^{2}(a_{1}+a_{2}^{CP}+f)-{\hat{\sigma }}_{LR,1}^{2}(s_{1})}-\sqrt{\eta_{22}}\cdot {\hat{\mu }}_{2}\sigma_{LR,2}(a_{2}^{CP}+f))}{\sqrt{\eta_{12}-\eta_{12}+\eta_{22}}}, \end{array}$$

where ${\hat{\mu }}_{k}=-\sqrt{n_{k}}\cdot \log\;(\hat{\omega })$ is the estimated drift. Plugging in the identities $n_{2}=a_{2}^{CP}\cdot r^{\prime }$ and the formulas for $\sigma_{LR,2}(a_{2}^{CP}+f)$ and $\sigma_{LR,1}(a_{1}+a_{2}^{CP}+f)$ given by (14) with updated values $v_{2}\to v_{2}^{\prime }$ and $r\to r^{\prime }$ , we can solve above equation (33) to obtain $a_{2}^{CP}$ . Note that the equation can not be solved if $\hat{\omega }\geq 1$ holds. In this case we define $a_{2}^{CP}\;:\;=\infty$ .

The revised length of accrual $a_{2}^{\prime }$ is finally chosen according to (30). We will provide R syntax to do so in the supplementary material.

## Example: A two–step log–rank test with futility criterion based on short–term survival rate

In this section, we illustrate application of our methodology using the example of a two-step log-rank test with binding futility criterion based on a short-term survival rates and sample size recalculation based on conditional power. Recall that the underlying null hypothesis is $H_{0}\;:\;S_{A}(s)=S_{B}(s)$ for all $0\leq s\leq s_{\max}$ for some prefixed $s_{\max}>0$ . The underlying physical units of s will be ”years”.

In general, our two-step test of $H_{0}$ depends on a set of design parameters that have to be fixed in advance:

(a) parameters $b_{0}$ , $u_{0}$ , $u_{1}$ , $u_{2}$ defining the rejection region acc. to (22),

(b) parameters $s_{0}$ and $s_{1}$ steering the amount of follow-up included into interim decision making,

(c) parameters $a_{1}$ , $a_{2}$ , f defining the initially planned lengths of stage one accrual, stage two accrual, and follow-up period

(d) parameters r , $v_{1}$ , $v_{2}$ defining the initial accrual rate, and stage-wise treatment arm allocation ratios

(e) weights $\eta_{11}$ , $\eta_{12}$ and $\eta_{22}$ of the stage-wise log-rank increments acc. to (20) and (14).

More specifically, let us assume that we aim for a two-step, Pocock-type log-rank test of $H_{0}$ with binding stopping for futility if the observed 6 months survival rate in the experimental arm is worse than in the standard arm. This futility condition is realized by choosing $b_{0}=0$ , $u_{0}=-\infty$ , and $s_{0}=0.5$ . The Pocock condition means choosing $u_{1}=u_{2}$ .^5^ Note that an uncountable number of alternative functional relationships between $u_{1}$ and $u_{2}$ could have been chosen. The difference $s_{1}$ - $s_{0}$ is the interval between the time when the short-term endpoint $B_{1}$ becomes known and the date of the interim analysis. For practical reasons, $s_{1}-s_{0}\geq 0$ should not be chosen too large. On the other hand, $s_{1}$ should be sufficiently large such that the interim log-rank statistic $Z_{1}$ is informative. In our exemplary setting, we consider $s_{1}=1$ as sensible choice. The parameters f and r are determined by the clinical frame conditions. Let us assume a desired follow-up period of f=2 years, and an annual overall accrual rate of r=75 . Also assume that we aim for equal randomization to both arm (i.e. $v_{1}=v_{2}=1$ ) as well as an interim analysis after half of the planned overall accrual period, i.e. $\pi \;:\;=a_{1}/(a_{1}+a_{2})=0.5$ . Finally, assume that we set a significance level of 5% , that we aim for a power of 80% if the true hazard ratio $\omega_{0}$ equals 2/3 (planning alternative hypothesis), and that there are exponentially distributed survival times with scale parameter of λ=1 to a good approximation in the standard therapy arm.

With these specifications, the parameters $u_{1}$ and $a_{1}$ remain as the only unknown from the parameters listed under a)-d). Whereas the weight $\eta_{11}$ is also fixed by above specifications, the weights $\eta_{12}$ and $\eta_{22}$ remain as functions in $a_{1}$ acc. to equation (20), since $s_{1}=1$ , $a_{1}+f_{1}=2\cdot a_{1}+f$ , $f_{1}=a_{1}+f$ . We are now in a position to determine the rejection region (see Section ‘The rejection region’) and to perform the initial sample-size calculation (see Section ‘Calculation of the critical bounds’). Using $b_{0}=0$ , $u_{0}=-\infty$ , $u_{1}=u_{2}$ and ρ acc to (8) the equations (22) and (26) may be solved simultaneously for the two remaining free parameters $u_{1}$ and $a_{1}$ . Doing so, yields a stage-one recruitment period length of $a_{1}=1.7$ years (corresponding to $n_{1}=r\cdot a_{1}=125$ patients), together with a stage-one critical boundary $u_{1}=2.18$ . On this basis, the weights may be calculated as $\eta_{11}=0.158$ , $\eta_{12}=0.247$ , $\eta_{22}=0.233$ using (20) and (14). To ensure that the rejection region does not depend on our initial planning assumptions regarding ρ , the value of the critical bound $u_{2}$ will be updated and ultimately fixed as described below at the time of the interim analysis, when an estimate of ρ becomes available.

After 1.7+0.5=2.3 years, instead of $B_{1}$ can be evaluated. Assume that we find a value of instead of $B_{1}=1.08>0=b_{0}$ , which concludes that the trial can continue (no stopping for futility). After 1.7+1.0=2.7 years, the interim log-rank statistic $Z_{11}$ becomes known and the interim analysis has to be performed. Let us assume that a test statistic of $Z_{11}=1.34<2.18=u_{1}$ is observed as well as an estimated hazard ratio of $\hat{\omega }=0.731$ . In this case the trial continuous to stage two and the sample-size can be adapted in the light of this new information.

In a first step we now estimate the covariance parameter ρ according to (24) in the light of the interim data. Assume that we find an estimated value of $\hat{\rho }=0.733$ . With this estimate we calculate the final value of the stage–two critical boundary $u_{2}$ by solving (25) with our estimate plugged in as ρ , and all remaining parameters as specified as above. Doing so yields in the value $u_{2}=2.17$ and ensures that the rejection region does not depend on our initial planing assumptions regarding ρ .

Having determined the final rejection region, let us now recalculate the sample-size such that a conditional power of $1-\beta_{2}=0.8$ is achieved, say, under the constraint that the overall accrual period is at least $a_{1}+s1=2.7$ years, but must not exceed $a_{\max}=5$ years. Notice that it is principally possible to adapt the recruitment rate r or the allocation ratio $v_{2}$ depending on $B_{1}$ or $Z_{11}$ at time of the interim analysis. For simplicity, we here assume that neither accrual rate nor allocation ratio shall be adapted, i.e. we choose $r^{\prime }=r$ and $v_{2}^{\prime }=v_{2}$ . In order to carry out sample size recalculation according to these specification, we first calculate the required length $a_{2}^{CP}$ of the second stage accrual period to realize the desired conditional power of $1-\beta_{2}=0.8$ . This can be done by solving equation (33) for the only remaining indeterminate $a_{2}^{CP}$ , which in our case yields $a_{2}^{CP}=3.0$ . To implement the constraint on the minimum and maximum length of accrual, the revised length $a_{2}^{\prime }$ of the second stage accrual period is finally chosen according to (30). With $a_{\max}=5$ , $a_{1}=1.7$ , $s_{1}=1$ equation (30) yields $a_{2}^{\prime }=a_{2}^{CP}=3.0$ , corresponding to $n_{2}=226$ patients in stage two.

Finally after 1.7+3.0+2.0=6.7 years after start of the trial, the final analysis is due. At this time the test statistics $Z_{12}$ and $Z_{22}$ become known. Assuming that $Z_{12}=1.67$ and $Z_{22}=3.14$ are observed, we finally obtain the final test statistic $Z_{2}^{*}$ according to (19)

$$Z_{2}^{*}=\frac{\sqrt{0.158}\cdot 1.34+\sqrt{0.247-0.158}\cdot 1.67+\sqrt{0.233}\cdot 3.14}{\sqrt{0.247+0.233}}=3.67>2.17=u_{2}^{\prime },$$

which concludes a successful trial with rejection of $H_{0}$ after stage two.

We will present an example design for a seamless phase II/III trial in detail in the supplemental material.

## Simulation

### Design of the main scenario

We consider testing the hypothesis formulated in equation (5) $H_{0}\;:\;S_{A}(s)=S_{B}(s)$ for all $0\leq s\leq s_{\max}$ using the two-step adaptive design presented in section 4.1.

In the context of the LOGGIC Europe trial, it was of interest to show a positive effect on the short term PFS-rate at an interim analysis to obtain the preliminary conditional marketing authorisation. Only with this conditional marketing authorization it was desired to continue recruitment of patients and to additionally test the effect on the long-term progression free survival.

More specifically, a design with rejection region of the form

$$R\;:\;=\{Z_{1}^{*}\geq u_{1}\}\cup \{B_{1}>0,Z_{1}^{*}<u_{1},Z_{2}^{*}\geq u_{2}\}$$

would have been of interest. Notice that we set the critical boundaries $b_{0}=0$ and $u_{0}=-\infty$ . We set $s_{0}=s_{1}=1.5$ , f=2 and π=0.5 . This corresponds to a two–step log–rank test with binding futility criterion based on the 18-months response rate.

The following frame conditions were chosen as the main scenario for this simulation study: Patients are allocated equally to both treatment arms (allocation ratio $v_{1}=v_{2}=1$ ). Survival times are Weibull distributed with scale parameter of m=1/log(2) and shape parameter k=1 , which corresponds to a scaled exponential distribution with median survival of 1 year. To study the performance of our algorithm we ran also simulations with shape parameters k=0.5 and k=2 . Planing was done under the planing alternative $S_{B}(s)=S_{A}{\left(s\right)}^{\omega_{1}}$ , where $\omega_{1}=2/3$ . We also ran simulations with $\omega_{1}=4/5$ . We let the true hazard ratio ω range between 0.5 and 1 in steps of 1/15 . The one sided type 1 error rate was set to α=0.025 and the desired power was set to 1−β=0.8 . We set the conditional power parameter $\beta_{2}$ such that it satisfies the equation

(34) $$P_{K_{1}}(Z_{1}^{*}\geq u_{1})+P_{K_{1}}(Z_{1}^{*}<u_{1},B_{1}\geq b_{0})\cdot (1-\beta_{2})=1-\beta .$$

**Figure 2.**
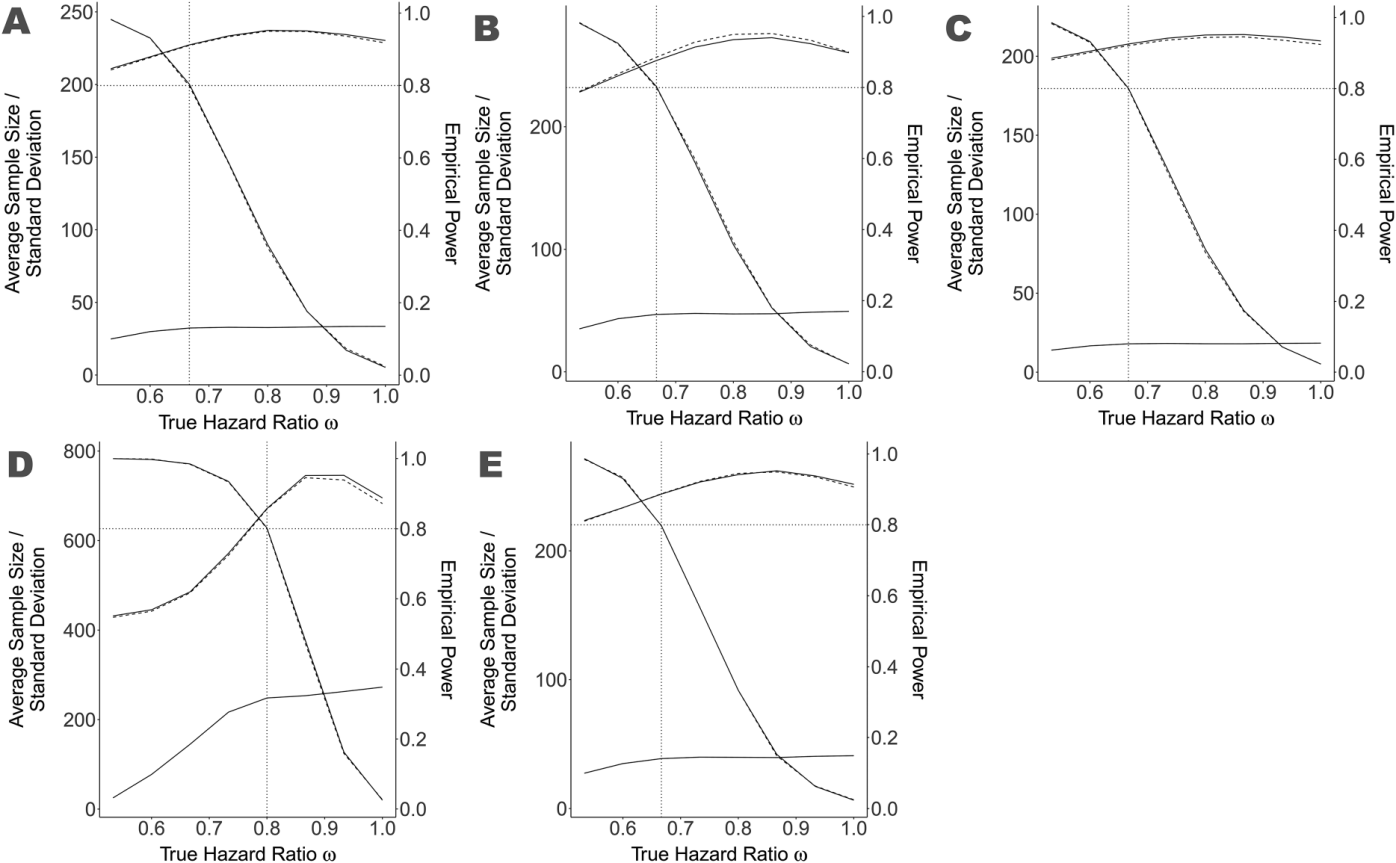
Average sample size, standard deviation of sample size and empirical power of the main scenario $\left(k=1,\omega_{1}=2/3,r=60,b_{0}=0,u_{0}=-\infty ,u_{1}=\infty \right)$ and some variations true hazard ratio ω ranging between 0.5 and 1.0, compared to a standard adaptive design with stop for futility. The solid lines represent our new methodology and the dashed lines the standard methodology, where the monotone decreasing lines starting at nearly 1 and ending by 0.025 represent the empirical power. The remaining upper lines show the average sample size and the lower lines the standard deviation of the sample size. Notice that the latter lines overlap considerably and are therefore difficult to distinguish. The vertical dotted line represents the value for ω used as planing alternative. The dotted, horizontal line represents the aimed power of 80 % . Figure A is the main scenario, Figure B the variation with k=0.5 , Figure C the variation with k=2.0 , Figure D the variation with ω=0.8 and Figure E is the variation Pocock boundaries. The value of the fine-tune parameter PF is presented in the table on the bottom right for each scenario variation.

This choice tries to stabilize the power of the whole trial despite the adaptation. The recruitment rate was set to r=60 . The maximal trial duration $a_{\max}$ was set as PF=1.5 times the duration of a corresponding single–step two–sample log–rank test.^1^ In some of our scenarios (Figure 2) we let the parameter PF vary in the set [1.25,1.75] as a fine-tuning parameter.

No loss to follow–up was assumed as well as block-randomization and uniform recruitment assumptions as required by theorem A2.

For each simulation the required recruitment period lengths of stage one $a_{1}$ and stage two $a_{2}$ were calculated according to section ‘Initial sample size calculation’. In our simulations we additionally distinguished between (i) a Pocock-type design with $u_{1}=u_{2}$ and (ii) a design without early stopping where $u_{1}\;:\;=\infty$ . Note that the critical bounds $b_{0}$ and $u_{1}$ have to be fixed in advance, whereas the value for $u_{2}$ is calculated according to equation (22) at the interim analysis, when the estimator $\hat{\rho }$ for ρ becomes available. Thus the theoretical equality ” $u_{1}=u_{2}$ ” in the Pocock setting is effectively only realized approximately.

With above values for r , $a_{1}$ , $a_{2}$ and f , the weights $\eta_{11}$ , $\eta_{12}$ , $\eta_{22}$ were calculated according to equations (20).

Then $r\cdot a_{1}$ patients were simulated as first stage patients, with preliminary censoring at study time $s_{1}$ , which represents the data we are allowed to use at the interim analysis. Based on this simulated data the interim statistics $Z_{1}^{*}$ , $B_{1}$ , $N_{1}$ , $\hat{\rho }$ and $\hat{\omega }$ were calculated.

The test statistics $Z_{1}^{*}$ and $B_{1}$ were then compared to the prefixed critical bounds $b_{0}$ and $u_{1}$ to determine whether early successful stopping or stopping for futility has occurred.

In the case of an ongoing trial, i.e. $B_{1}>b_{0}$ and $Z_{1}^{*}<u_{1}$ , the critical bound $u_{2}$ is obtained by solving equation (22) with the estimator $\hat{\rho }$ plugged in. Additionally, the required recruitment period length of stage two $a_{2}^{CP}$ was calculated such that a conditional power of $1-\beta_{2}$ is achieved under the revised planing alternative hypothesis $K_{1}^{\prime }\;:\;S_{B}=S_{A}^{\hat{\omega }}$ corresponding to the observed hazard ratio $\hat{\omega }$ . The actual recruitment period length of stage two patients $a_{2}^{\prime }$ was then updated as stated in (30), to stipulate the boundary conditions.

We then proceeded (i) to simulate $a_{2}^{\prime }\cdot r$ patients of stage two and (ii) to update the censoring date of stage one patients to calendar time $a^{\prime }+f$ .

Finally the test-statistic $Z_{2}^{*}$ was calculated according to (19) and compared to the critical bound $u_{2}$ derived at the interim analysis to obtain the final test decision.

The above presented simulation algorithm was run 10,000 times for each scenario.

### Results

The simulation results are presented briefly in Table 1. Reassuringly the designs hold the aimed significance level of 2.5% , even in the small sample size case. Note that with 10,000 simulations per scenario the estimated accuracy of our type one error rate estimator given through 95% -confidence intervals is ±0.31% . Accordingly in no scenario the empirical type I error rate exceeded the aimed significance level of 2.5% in a statistically noticeable way.

**Table 1.** Empirical type I error rate and power in the simulation scenarios. Empirical type I error rate (TOE) was obtained from simulations where the true hazard ratio ω=1 . Empirical power was obtained by simulations where the true hazard ratio equals the planing hazard ratio $\left(\omega =\omega_{1}\right)$ . For further simulation details see section 6.

k	ω	$u_{1}$	Average n	Emp. TOE	Emp. power
0.5	2/3	∞	279.453	0.027	0.838
		Pocock	283.680	0.024	0.817
	4/5	∞	738.387	0.024	0.798
		Pocock	755.432	0.025	0.804
1.0	2/3	∞	249.870	0.026	0.839
		Pocock	256.583	0.025	0.815
	4/5	∞	671.721	0.025	0.793
		Pocock	690.295	0.026	0.799
2.0	2/3	∞	232.490	0.024	0.847
		Pocock	239.399	0.024	0.814
	4/5	∞	651.614	0.026	0.793
		Pocock	672.480	0.026	0.796

The empirical power however shows a little more variation. This is due to the fact, that the initial sample size calculation does not factor in the randomness introduced by $\hat{\omega }$ , which effects the sample size recalculation based on conditional power. This is a well-known effect of such adaptive designs.

*Main simulation scenario.* The strength of adaptive designs is undoubtedly the possibility for correction when the initial planning assumptions seem to be wrong. When the treatment effect is small one can stop for futility or increase the sample size to hold on the desired power. Conversely when the treatment effect is larger than expected one can decrease the sample size while still holding the desired power.

We simulated our main scenario ( $k=1,\omega_{1}=2/3,r=60,u_{1}=\infty )$ with some variations. We used the parameter PF∈[1.25,1.75] as a fine-tuning parameter to level out the variation introduced by $\hat{\omega }$ and to match the aimed 80% power quite exactly. The choice of these fine-tuning parameter is presented in the table within Figure 2.

We compare our test algorithm with a standard adaptive design based on the standard methodology by Wassmer.^3^ To assure comparability we implemented a futility stop, when the short term log-rank test $Z_{1}$ based on the first half of patients shows a negative result. More specifically in the non Pocock designs, we compared our design to an adaptive design with rejection region

(35) $$R_{\mathrm{simple},1}\;:\;=\{Z_{1}\geq 0,Z_{2}^{*}\geq \Gamma_{1}\},$$

where $\Gamma_{1}$ is chosen such that $P_{H_{0}}(R_{\mathrm{simple},1})=\alpha$ . In the Pocock scenario we compared our design to a design with rejection region

(36) $$R_{\mathrm{simple},2}\;:\;=\{Z_{1}\geq \Gamma_{2}\}\cup \{0\leq Z_{1}<\Gamma_{2},Z_{2}^{*}\geq \Gamma_{2}\},$$

where $\Gamma_{2}$ is chosen such that $P_{H_{0}}(R_{\mathrm{simple},2})=\alpha$ . These are rejection regions, which can be used within the methodology of Wassmer and are included in our methodology.

We set the required sample size such that the standard design also holds the desired power of 1−β=80% under the planing hypothesis $K_{1}$ .

The operating characteristic of our test algorithm in the main simulation scenario $\left(k=1,\omega_{1}=2/3,u_{1}=\infty \right)$ is presented in Figure 2 together with some variations of the scenario.

Across all scenario variations, the power and sample-size performance of our test statistic fits the performance of the standard methodology quite well.

In the main scenario the mean sample-size difference between the standard methodology and our new methodology is 0.74 % at maximum. Under the planing hypothesis the maximal increase of the mean sample-size across all scenario variations was 0.5 % , while in some cases the new design reduced the mean sample-size about 1.0% .

This suggests the use of easily interpretable survival rate differences as an interesting option for interim decision making in survival trials.

By using various Weibull shape parameters, planning hypothesis and design types we assured that the performance consistency is not dependant on our specific scenario assumptions.

## Discussion

The confirmatory adaptive two–step log–rank test proposed here extends the one proposed by Wassmer.^3^ Whereas the test proposed by Wassmer essentially only allows the use of the interim log–rank statistic for data–dependent design modifications, our approach allows simultaneous use of the interim log–rank statistic and observed differences in cumulative hazard rates at time $s_{0}$ for interim decision making, while avoiding those problems arising with methods based on patient wise separation.^6–8^ Next to an adaptation of sample size, our approach also allows modification of the allocation ratio between the treatment arms or the recruitment rate, which neither has been described by Wassmer^3^ nor Jenkins.^6^ This is of importance when thinking about application of our methodology in a multiarm, multistage setting. Even though the focus of this paper was on a trial design with two treatment arms and two analyses, the generalization to more than two arms and more than two analyses is straightforward using the methodology described by Hommel et al.^9^

Our adaptive two–step log–rank test exploits the independent increments structure of the limiting Gaussian process of the joint bivariate process defined by the log–rank statistic and the Nelson–Aalen difference at some time $s_{0}$ . Therefore, we emphasize that the full use of arbitrary interim data for design modifications is still not admissible here.^4^ However, our approach makes provision for the simultaneous use of (i) the interim log–rank statistic and (ii) differences in cumulative hazard rates at an arbitrary time $s_{0}$ .

The calculation of rejection regions and sample size formulas were based on distributional approximation of the bivariate test statistic in the large sample limit. Our methodology used mild regularity assumptions as well as the proportional hazards assumption. It is well known, that the log–rank test is less efficient and its distribution depends on the distribution of censoring times, when the proportional hazards assumption is violated.^10^ This is likely to be inherited by our method. The small sample properties were studied by simulations. The validity of the proposed design does not depend on specific model assumptions underlying these simulations such as exponentially distributed survival times. In view of the flexibility offered by our approach, however, applicants are recommended to assess different choices of design parameters in order to identify those parameter constellations with best operating characteristics as compared to a standard single–step two–sample log–rank test. For this purpose, we provide an R program in the supplemental material that enables easy assessment of operating characteristics and thus optimal calibration of the design parameters in a specific trial setting. The R program also underlies our simulation.

## Supplemental Material

sj-R-1-smm-10.1177_09622802211043262 - Supplemental material for Adaptive group sequential survival comparisons based on log-rank and pointwise test statistics Click here for additional data file. Supplemental material, sj-R-1-smm-10.1177_09622802211043262 for Adaptive group sequential survival comparisons based on log-rank and pointwise test statistics by Jannik Feld, Andreas Faldum and Rene Schmidt in Statistical Methods in Medical Research

sj-R-2-smm-10.1177_09622802211043262 - Supplemental material for Adaptive group sequential survival comparisons based on log-rank and pointwise test statistics Click here for additional data file. Supplemental material, sj-R-2-smm-10.1177_09622802211043262 for Adaptive group sequential survival comparisons based on log-rank and pointwise test statistics by Jannik Feld, Andreas Faldum and Rene Schmidt in Statistical Methods in Medical Research

sj-R-3-smm-10.1177_09622802211043262 - Supplemental material for Adaptive group sequential survival comparisons based on log-rank and pointwise test statistics Click here for additional data file. Supplemental material, sj-R-3-smm-10.1177_09622802211043262 for Adaptive group sequential survival comparisons based on log-rank and pointwise test statistics by Jannik Feld, Andreas Faldum and Rene Schmidt in Statistical Methods in Medical Research

## Appendix A

We will now deduce the distributional approximation presented in (8). The proofs presented here are formulated for a single-step design. However, the extension to a multi-step design is straight forward using the independent increments structure. We therefore drop the stage indices k for notational simplicity.

It is well known that for a patient i from treatment group x=A,B ,

(37)
$$M_{i}(s)\;:\;=D_{i}(s)-\int_{0}^{s}I(T_{i}\wedge C_{i}\geq u)\lambda_{x}(u)du$$

is an $F_{s}$ –martingale.^11^ In particular, with $M_{x}(s)\;:\;=\sum_{i\in N_{x}}M_{i}(s)$ and for any $F_{s}$ –adapted left–continuous process H(s) ,

$$\begin{array}{rl} (H∙M_{x})(s): & =\int_{0}^{s}H(u)dM_{x}(u)\\ & =\int_{0}^{s}H(u)dD_{x}(u)-\int_{0}^{s}H(u)Y_{x}(u)\lambda_{x}(u)du \end{array}$$

is an $F_{s}$ –martingale with optional and predictable covariation process^12^

$$\begin{array}{rl} \left[H∙M_{x}](s\right) & =\int_{0}^{s}H^{2}(u)dD_{x}(u),\\ \left\langle H∙M_{x}\rangle (s\right) & =\int_{0}^{s}H^{2}(u)Y_{x}(u)\lambda_{x}(u)du. \end{array}$$

We aim for the joint distribution of the weighted two–sample log–rank statistic, which has the integral representation

$$LR(s)=\int_{0}^{s}\frac{L(u)}{Y_{A}(u)}dN_{A}(u)-\int_{0}^{s}\frac{L(u)}{Y_{B}(u)}dN_{B}(u)$$

and the difference of the group–wise Nelsen–Aalen estimates

$$\Delta (s)=\int_{0}^{s}\frac{J_{A}(u)}{Y_{A}(u)}dN_{A}(u)-\int_{0}^{s}\frac{J_{B}(u)}{Y_{B}(u)}dN_{B}(u)$$

as $F_{s}$ –adapted processes, i.e. we aim for the distribution of the bivariate process

$$\begin{array}{rl} \Psi (s) & \;:\;=\left(\begin{array}{c} n^{-1/2}LR(s)\\ n^{1/2}\Delta (s) \end{array}\right). \end{array}$$

Introducing the bivariate drift process

$$\begin{array}{rl} \mu (s) & \;:\;=\left(\begin{array}{c} n^{-1/2}\int_{0}^{s}L(u)\{\lambda_{A}(u)-\lambda_{B}(u)\}du\\ n^{1/2}\int_{0}^{s}\{J_{A}(u)\lambda_{A}(u)-J_{B}(u)\lambda_{B}(u)\}du \end{array}\right), \end{array}$$

it follows from (37) that

$$\begin{array}{r} \left(\begin{array}{c} M^{LR}(s)\\ M^{\Delta }(s) \end{array}\right)\;:\;=M(s)\;:\;=\Psi (s)-\mu (s) \end{array}$$

is a bivariate mean–zero $F_{s}$ –martingale. Since $[M_{x}]=N_{x}$ and $[M_{A},M_{B}]=0$ , the optional covariation matrix [M](s) of M has components

$$\begin{array}{rl} \left[M^{LR}](s\right) & =n^{-1}\int_{0}^{s}\frac{L{\left(u\right)}^{2}}{Y_{A}{\left(u\right)}^{2}}dN_{A}(u)+n^{-1}\int_{0}^{s}\frac{L{\left(u\right)}^{2}}{Y_{B}{\left(u\right)}^{2}}dN_{B}(u),\\ \left[M^{LR},M^{\Delta }](s\right) & =\int_{0}^{s}\frac{L(u)}{Y_{A}{\left(u\right)}^{2}}dN_{A}(u)+\int_{0}^{s}\frac{L(u)}{Y_{B}{\left(u\right)}^{2}}dN_{B}(u),\\ \left[M^{\Delta }](s\right) & =n\int_{0}^{s}\frac{J_{A}(u)}{Y_{A}{\left(u\right)}^{2}}dN_{A}(u)+n\int_{0}^{s}\frac{J_{B}(u)}{Y_{B}{\left(u\right)}^{2}}dN_{B}(u). \end{array}$$

Since $\langle M_{x}\rangle (s)=\int_{0}^{s}Y_{x}(u)\lambda_{x}(u)du$ and $\langle M_{A},M_{B}\rangle =0$ , the predictable covariation matrix ⟨M⟩(s) of M has components

$$\begin{array}{rl} \left\langle M^{LR}\rangle (s\right) & =n^{-1}\int_{0}^{s}\frac{L{\left(u\right)}^{2}}{Y_{A}(u)}\lambda_{A}(u)du+n^{-1}\int_{0}^{s}\frac{L{\left(u\right)}^{2}}{Y_{B}(u)}\lambda_{B}(u)du,\\ \left\langle M^{LR},M^{\Delta }\rangle (s\right) & =\int_{0}^{s}\frac{L(u)}{Y_{A}(u)}\lambda_{A}(u)du+\int_{0}^{s}\frac{L(u)}{Y_{B}(u)}\lambda_{B}(u)du,\\ \left\langle M^{\Delta }\rangle (s\right) & =n\int_{0}^{s}\frac{J_{A}(u)}{Y_{A}(u)}\lambda_{A}(u)du+n\int_{0}^{s}\frac{J_{B}(u)}{Y_{B}(u)}\lambda_{B}(u)du. \end{array}$$

Above equations are easily checked (see Aalen et al.^13^ Sec. 2.2.5). On this basis we may deduce the distributional properties of the bivariate process Ψ(s)=M(s)+μ(s) in the large sample limit, as stated in the following theorems. The proofs of the theorems A1, A2 and equations (13) and (14) are presented after some additional results, which we need.

Theorem A1
*Fix* $s_{\max}>0$ and assume that the hazard functions $\lambda_{A}$ and $\lambda_{B}$ are bounded on the interval $\left[0,s_{\max}\right]$ and $P(C_{i}>s_{\max})>0$ . Assume furthermore that the treatment group allocation is done by block randomisation i.e. there exists a constant BL∈N , that for all n∈N it holds

$$\|\#N_{A}^{n}-\left\lfloor \frac{1}{1+v}\cdot n\right\rfloor \|\vee \|\#N_{B}^{n}-\left\lfloor \frac{v}{1+v}\cdot n\right\rfloor \|<BL.$$

Then for all $s\in [0,s_{\max}]$ the limit $\Sigma (s)\;:\;={\text{plim}}_{n\to \infty }[M](s)$ exists and there is a Gaussian mean zero martingale $M^{\infty }(s)$ with covariance matrix Σ(s) s.t.

$$M\frac{D}{n\to \infty }M^{\infty }.$$

In particular, the following distributional approximation holds:

$$\begin{array}{rl} \left(i\right) & M(s_{2})-M(s_{1})\mathrm{and}\;M(s_{1})\mathrm{are}\;\mathrm{independent}\;\mathrm{for}\;\mathrm{all}\;0\leq s_{1}\leq s_{2}\leq s_{\max}.\\ \left(ii\right) & M(s)∼N(0,\Sigma (s))\;\mathrm{for}\;\mathrm{all}\;s\in [0,s_{\max}].\\ \left(iii\right) & M(s_{2})-M(s_{1})∼N(0,\Sigma (s_{2})-\Sigma (s_{1}))\mathrm{for}\;\mathrm{all}\}0\leq s_{1}\leq s_{2}\leq s_{\max}. \end{array}$$

Theorem A2
Assume the conditions from theorem 1. Then, under the contiguous alternatives $\Lambda_{B}(s)=\omega_{n}\Lambda_{A}(s)$ with $\omega_{n}\;:\;=e^{-n^{-1/2}\gamma }$ for γ≥0 , we have

$$\begin{array}{rl} \Sigma (s) & =\left(\begin{array}{cc} \sigma_{LR}^{2}(s) & \Lambda_{A}(s)\\ \Lambda_{A}(s) & \sigma_{\Delta }^{2}(s) \end{array}\right)\mathrm{and}\;\lim_{n\to \infty }\mu (s)=\left(\begin{array}{c} \gamma \cdot \sigma_{LR}^{2}(s)\\ \gamma \cdot \Lambda_{A}(s) \end{array}\right), \end{array}$$

for some continuous variance functions $\sigma_{LR}^{2}(s)$ and $\sigma_{\Delta }^{2}(s)$ . More explicit we have $\sigma_{LR}(s)=\sqrt{\pi (s)}\frac{\sqrt{v}}{1+v}$ , where $\pi (s)\;:\;={\text{plim}}_{n\to \infty }N(s)/n$ . In particular the limit π(s) exists. Since Ψ(s)=M(s)+μ(s) , we conclude that the processes in (38) below have independent increments with the following distributional large sample approximation:

(38)
$$\begin{array}{rl} & \left(\begin{array}{c} n^{-1/2}LR(s)\\ n^{1/2}\Delta (s) \end{array}\right)\overset{D}{\approx }\;N(\left(\begin{array}{c} \gamma \cdot \sigma_{LR}^{2}(s)\\ \gamma \cdot \Lambda_{A}(s) \end{array}\right),\left(\begin{array}{cc} \sigma_{LR}^{2}(s) & \Lambda_{A}(s)\\ \Lambda_{A}(s) & \sigma_{\Delta }^{2}(s) \end{array}\right)),\\ & \left(\begin{array}{c} \frac{n^{-1/2}LR(s)}{\sqrt{\left[M^{LR}](s\right)}}\\ \frac{n^{1/2}\Delta (s)}{\sqrt{\left[M^{\Delta }](s\right)}} \end{array}\right)\overset{D}{\approx }\;N(\left(\begin{array}{c} -\sqrt{n}\log\;(\omega )\cdot \sigma_{LR}(s)\\ -\sqrt{n}\log\;(\omega )\cdot \frac{\Lambda_{A}(s)}{\sigma_{\Delta }(s)} \end{array}\right),\left(\begin{array}{cc} 1 & \frac{\Lambda_{A}(s)}{\sigma_{LR}(s)\sigma_{\Delta }(s)}\\ \frac{\Lambda_{A}(s)}{\sigma_{LR}(s)\sigma_{\Delta }(s)} & 1 \end{array}\right)). \end{array}$$

Using the independence of increments we get for $0<s_{0}\leq s_{1}\leq s_{\max}$

$$\left(\begin{array}{c} \frac{n^{-1/2}LR(s_{1})}{\sqrt{\left[M^{LR}](s_{1}\right)}}\\ \frac{n^{1/2}\Delta (s_{0})}{\sqrt{\left[M^{\Delta }](s_{0}\right)}} \end{array}\right)\overset{D}{\approx }\;N(\left(\begin{array}{c} -\sqrt{n}\log\;(\omega )\cdot \sigma_{LR}(s_{1})\\ -\sqrt{n}\log\;(\omega )\cdot \frac{\Lambda_{A}(s_{0})}{\sigma_{\Delta }(s_{0})} \end{array}\right),\left(\begin{array}{cc} 1 & \frac{\Lambda_{A}(s_{0})}{\sigma_{LR}(s_{1})\sigma_{\Delta }(s_{0})}\\ \frac{\Lambda_{A}(s_{0})}{\sigma_{LR}(s_{1})\sigma_{\Delta }(s_{0})} & 1 \end{array}\right)),$$

which is the distributional approximation we stated in (8). To prove our theorems we need some additional results.

Proposition A3 Let $(X_{n}(s){)}_{n\in N}$ be a sequence of stochastic processes, f a borel measurable function and $s_{\max}>0$ with

(1) $X_{n}(s)\frac{P}{n\to \infty }f(s),$
(2) $\int_{0}^{s_{\max}}|f(s)|<\infty$ ,

which also satisfy one of the following conditions.

- It exists a constant c>0 , that for all n∈N and for all $s\in [0,s_{\max}]$ it holds
  $$|X_{n}(s)|<c\;a.s..$$
- It exists a sequence of stochastic processes $({\tilde{X}}_{n}(s){)}_{n\in N}$ , a function h and an integrable function g with

- $\left|{\tilde{X}}_{n}(s)\right|$ is monotone in s for all n∈N a.s.,
- $\left|{\tilde{X}}_{n}(s)|\frac{P}{n\to \infty }h(s\right)$ for all $s\in [0,s_{\max}]$ ,
- $\left|X_{n}(s)|\leq |{\tilde{X}}_{n}(s)\cdot g(s)\right|$ for all $s\in [0,s_{\max}]$ a.s..

Then it holds

$$\int_{0}^{s}X_{n}(u)du\frac{P}{n\to \infty }\int_{0}^{s}f(u)du\;\mathrm{for}\;\mathrm{all}\;s\in [0,s_{\max}].$$

Proof. We will show that conditions (1),(2) and (a) are sufficient to satisfy the preconditions of Hellands proposition^12^ Prop. II.5.2 and that conditions (1),(2) and (b) are sufficient to satisfy the preconditions of Gills proposition^12^ Prop. II.5.3 . Assume conditions (1),(2) and (a) are satisfied. Then for any $c^{\prime }>c$ it holds
$1_{\left\{|X_{n}(s)|>c^{\prime }\right\}}=0$ a.s. and furthermore

$$\underset{n\in N}{\sup}E\left[|X_{n}(s)|1_{\left\{|X_{n}(s)|>c^{\prime }\right\}}\right]=0.$$

With k(s):=c it holds

$$E\left[|X_{n}(s)|\right]\leq E[c]=c=k(s).$$

Therefore the preconditions of Hellands proposition are satisfied. Assume now that conditions (1),(2) and (b) are satisfied. Without loss of generality we can assume, that $\left|{\tilde{X}}_{n}(s)\right|$ is monotone increasing in s . Otherwise we transition to $\left|{\tilde{X}}_{n}(s_{\max}-s)\right|$ . For arbitrary δ>0 we choose some arbitrary but fixed ϵ>0 and define $k_{\delta }(s)\;:\;=|\{h(s_{\max})+\epsilon \}\cdot g(s)|$ . It holds

$$\begin{array}{rl} & P(|X_{n}(s)|\leq k_{\delta }(s)\mathrm{for}\;\mathrm{all}\;s\in [0,s_{\max}])\\ = & P(|X_{n}(s)|\leq |\{h(s_{\max})+\epsilon \}\cdot g(s)|\mathrm{for}\;\mathrm{all}\;s\in [0,s_{\max}])\\ \geq & P(|{\tilde{X}}_{n}(s)\cdot g(s)|\leq |\{h(s_{\max})+\epsilon \}\cdot g(s)|\mathrm{for}\;\mathrm{all}\;s\in [0,s_{\max}])\\ \geq & P(|{\tilde{X}}_{n}(s)|\leq |\{h(s_{\max})+\epsilon \}|\mathrm{for}\;\mathrm{all}\;s\in [0,s_{\max}])\\ = & P(|{\tilde{X}}_{n}(s_{\max})|\leq |\{h(s_{\max})+\epsilon \}|)\frac{}{n\to \infty }1>1-\delta . \end{array}$$

In the last equality we used, that $\left|{\tilde{X}}_{n}(s)\right|$ is monotone increasing in s . The convergence holds because of the convergence of $\left|{\tilde{X}}_{n}(s)\right|$ in probability. Above inequality yields in the preconditions of Gills proposition.  □

Lemma A4 (**Simple weak law of large numbers**) Let $(\mu_{n}{)}_{n\in N}$ be a sequence in [0,1] with $\lim_{n\to \infty }\mu_{n}=\mu \in [0,1]$ and let $(X_{i}^{\left(n\right)}{)}_{i=1,\ldots ,n}$ be a sequence of independent *Ber* $\left(\mu_{n}\right)$ distributed random variables. Then it holds

$$\frac{\sum_{i=1}^{n}X_{i}^{\left(n\right)}}{n}\underset{n\to \infty }{\;⟶}\mu .$$

Proof. We will proof the convergence in probability by showing, that the convergence holds in $L^{2}$ . Define $S_{n}\;:\;=\sum_{i=1}^{n}X_{i}^{\left(n\right)}$ , then it holds

$$\begin{array}{rl} E\left[{\left(\frac{S_{n}}{n}-\mu \right)}^{2}\right]= & E\left[{\left(\frac{S_{n}}{n}-\mu_{n}+\mu_{n}-\mu \right)}^{2}\right]\\ = & E\left[{\left(\frac{S_{n}}{n}-\mu_{n}\right)}^{2}\right]+2E\left[\left(\frac{S_{n}}{n}-\mu_{n}\right)\left(\mu_{n}-\mu \right)\right]+E\left[{\left(\mu_{n}-\mu \right)}^{2}\right]\\ = & \frac{1}{n^{2}}\mathrm{Var}(S_{n})+2E\left[\frac{S_{n}}{n}-\mu_{n}\right]\cdot \left(\mu_{n}-\mu \right)+{\left(\mu_{n}-\mu \right)}^{2}\\ = & \frac{1}{n^{2}}n\mu_{n}(1-\mu_{n})+{\left(\mu_{n}-\mu \right)}^{2}\to 0 \end{array}$$

 □

Lemma A5 (**Weak law of large numbers**) Let $({\#}^{n}{)}_{n\in N}$ be an infinite sequence of N -valued, monotone in n∈N increasing random variables, which satisfy the following conditions

- $\forall n\in N\;:\;{\#}^{n}\leq n$ ,
- $\exists c\in [0,1]\;:\;\frac{{\#}^{n}}{n}\underset{n\to \infty }{\;⟶}c$ ,
- $\exists BL\in N\;:\;\forall n\in N\;:\;\|{\#}^{n}-\lfloor cn\rfloor \|<BL$ .

Let furthermore $(\mu_{n}{)}_{n\in N}$ be a sequence in [0,1] with $\lim_{n\to \infty }\mu_{n}=\mu \in [0,1]$ and $(X_{i}^{\left(n\right)}{)}_{i=1,\ldots ,n}$ a sequence of independent *Ber* $\left(\mu_{n}\right)$ distributed random variables. Then it holds

$$\frac{\sum_{i=1}^{{\#}^{n}}X_{i}^{\left(n\right)}}{n}\underset{n\to \infty }{\;⟶}c\cdot \mu .$$

Proof. We will show the convergence in $L^{2}$ . Define $Z_{n}\;:\;=\sum_{i=1}^{{\#}^{n}}X_{i}^{\left(n\right)}$ and $S_{n}\;:\;=\sum_{i=1}^{\left\lfloor cn\right\rfloor }X_{i}^{\left(n\right)}$ . Then it holds

$$\begin{array}{rl} E\left[{\left(\frac{Z_{n}}{cn}-\mu \right)}^{2}\right]= & E\left[{\left(\frac{Z_{n}}{cn}-\frac{S_{n}}{cn}+\frac{S_{n}}{cn}--\mu \right)}^{2}\right]\\ = & E\left[{\left(\frac{Z_{n}-S_{n}}{cn}\right)}^{2}\right]+2E\left[\left(\frac{Z_{n}-S_{n}}{cn}\right)\left(\frac{S_{n}}{cn}-\mu \right)\right]+E\left[{\left(\frac{S_{n}}{cn}-\mu \right)}^{2}\right]. \end{array}$$

The first summand satisfies the inequality

$${\left|\frac{Z_{n}-S_{n}}{cn}\right|}^{2}\leq {\left|\frac{{\#}^{n}-\lfloor cn\rfloor }{cn}\right|}^{2}\leq {\left|\frac{BL}{cn}\right|}^{2}\underset{n\to \infty }{\;⟶}0.$$

For the second summand it holds

$$\begin{array}{r} 2E\left[\left(\frac{Z_{n}-S_{n}}{cn}\right)\left(\frac{S_{n}}{cn}-\mu \right)\right]\leq 2E\left[\left|\frac{Z_{n}-S_{n}}{cn}\right|\left|\frac{S_{n}}{cn}-\mu \right|\right]\\ \leq 2\frac{BL}{cn}E\left[\left|\frac{S_{n}}{cn}-\mu \right|\right]\underset{n\to \infty }{\;⟶}0. \end{array}$$

For the convergence we used, that the second factor is bounded. The last summand vanishes analogously to the proof of prior lemma in the limit. Therefore the whole sum converges to 0. Multiplying both sides with c concludes the assertion.  □

*Proof of theorem A1*. We want to show, that ${\mathrm{plim}}_{n\to \infty }\langle M\rangle (s)$ exists. For this purpose we first take a closer look at the random variables $Y_{A}^{n}(u)$ and $Y_{B}^{n}(u)$ for some fixed $u\in R^{+}$ . If patient i∈N is under treatment A we use the notation $P_{A}\;:\;=P$ (analogously $P_{B}\;:\;=P$ ) to emphasize the stochastic influence of the treatment. It holds

$$Y_{A}^{n}(u)=\sum_{i\in N_{A}^{n}}1_{\left\{T_{i}>u,C_{i}>u\right\}}$$

and furthermore

$$P_{A}(T_{i}>u,C_{i}>u)=P_{A}(T_{i}>u)\cdot P(C_{i}>u)=S_{A}(u)\cdot P(C_{i}>u).$$

In the first equation we used the independence of $T_{i}$ and $C_{i}$ . With the independence of patients, it follows

$$Y_{A}^{n}(u)∼\mathrm{Bin}\left(\#N_{A}^{n},S_{A}(u)\cdot P(C_{i}>u)\right),$$

analogously it follows, that

(39)
$$Y_{B}^{n}(u)∼\mathrm{Bin}\left(\#N_{B}^{n},S_{B}(u)\cdot P(C_{i}>u)\right).$$

Because of block randomisation, the convergences $\frac{\#N_{A}^{n}}{n}\overset{P}{\underset{n\to \infty }{\;⟶}}\frac{1}{1+v}$ and $\frac{\#N_{B}^{n}}{n}\overset{P}{\underset{n\to \infty }{\;⟶}}\frac{v}{1+v}$ hold and we can apply the weak law of large numbers to get the convergences

(40)
$$\begin{array}{rl} \frac{Y_{A}^{n}(u)}{n} & \underset{n\to \infty }{\;⟶}\frac{1}{1+v}\cdot S_{A}(u)\cdot P(C_{i}>u)=\;:\;y_{A}(u),\\ \frac{Y_{B}^{n}(u)}{n} & \underset{n\to \infty }{\;⟶}\frac{v}{1+v}\cdot S_{B}(u)\cdot P(C_{i}>u)=\;:\;y_{B}(u). \end{array}$$

For the sake of readability we introduce the Notation −A:=B and −B:=A . Then ⟨M⟩(s) has components

(41)
$$\left\langle M_{n}^{\Delta }\right\rangle (s)=\sum_{x=A,B}n\int_{0}^{s}\frac{J_{x}^{n}(u)}{Y_{x}^{n}(u)}\lambda_{x}(u)du,$$

(42)
$$\left\langle M_{n}^{LR}\right\rangle (s)=\sum_{x=A,B}n^{-1}\int_{0}^{s}\frac{Y_{x}^{n}(u)\cdot Y_{-x}^{n}{\left(u\right)}^{2}}{\{Y_{x}^{n}(u)+Y_{-x}^{n}(u){\}}^{2}}\lambda_{x}(u)du,$$

(43)
$$\left\langle M_{n}^{\Delta },M_{n}^{LR}\right\rangle (s)=\sum_{x=A,B}\int_{0}^{s}\frac{Y_{-x}^{n}(u)}{Y_{x}^{n}(u)+Y_{-x}^{n}(u)}\lambda_{x}(u)du.$$

The limit in probability of the integrals can be computed with use of proposition A3. The integrands in (41) satisfy the preconditions (b) via

$${\tilde{X}}_{x}^{n}(u)=n\cdot \frac{J_{x}^{n}(u)}{Y_{x}^{n}(u)}+n\cdot 1_{\left\{J_{x}^{n}(u)=0\right\}}\overset{P}{\underset{n\to \infty }{\;⟶}}c_{x}\frac{1}{y_{x}(u)}\;\mathrm{and}\;g(u)=\lambda_{x}(u),$$

with $c_{A}=(1+v)$ and $c_{B}=(1+v)/v$ . Note that the convergence holds because of Slutsky’s theorem and equation (40). The integrands in (42) and (43) satisfy the precondition (a) of proposition A3, because they are bounded by $\underset{u\in [0,s_{\max}]}{\sup}\lambda_{x}(u)<\infty$ . It follows in probability as n→∞

- $\langle M_{n}^{LR}\rangle (s)\to \sum_{x=A,B}\int_{0}^{s}\frac{y_{x}(u)y_{-x}{\left(u\right)}^{2}}{\{y_{x}(u)+y_{-x}(u){\}}^{2}}\lambda_{x}(u)du$ ,
- $\langle M_{n}^{\Delta },M_{n}^{LR}\rangle (s)\to \sum_{x=A,B}\int_{0}^{s}\frac{y_{x}(u)}{y_{x}(u)+y_{-x}(u)}\lambda_{x}(u)du$ ,
- $\left\langle M_{n}^{\Delta }\rangle (s)\to \sum_{x=A,B}\int_{0}^{s}\frac{1}{y_{x}(u)}\lambda_{x}(u)du.(44\right)$ .

Moreover, the jumpsize of M(s) is of order $n^{-1/2}$ (and thus vanishes in the limit n→∞ ), because $N_{x}$ has jumpsize 1 and $Y_{x}$ is of order n . Thus, by a multivariate version of Rebolledo’s martingale central limit theorem^12^ (theorem II.5.1), ${\mathrm{plim}}_{n\to \infty }[M](s)$ exists and coincides with ${\mathrm{plim}}_{n\to \infty }\langle M\rangle (s)$ , and $[M(s){]}_{s\geq 0}$ converges to a mean zero Gaussian process $[M^{\left(\infty \right)}(t){]}_{t\geq 0}$ with independent increments and covariance matrix $\Sigma (s)=\;:\;{\mathrm{plim}}_{n\to \infty }[M](s)$ .  □

*Proof of theorem A2*. Let $y(u)\;:\;=(1+v)\cdot y_{A}(u)$ , with $y_{A}$ as in (40). Analogously to (39) it follows, that

$$Y_{B}^{n}(u)∼\mathrm{Bin}\left(\#N_{B}^{n},S_{A}^{\omega_{n}}(u)\cdot P(C_{i}>u)\right).$$

Using the weak law of large numbers, we therefore conclude

(45)
$$\frac{Y_{B}^{n}(u)}{n}\underset{n\to \infty }{\;⟶}\frac{v}{1+v}\cdot S_{A}(u)\cdot P(C_{i}>u)=v\cdot y_{A}(u).$$

We will now calculate the limit π and thus show its existence. Recalling that
$n^{-1/2}M_{A}(s)\overset{D}{\to }N(0,1)$ , we have in the limit n→∞ in probability

(46)
$$\begin{array}{rl} \frac{N(s)}{n} & =\frac{1}{n}M(s)+\frac{1}{n}\int_{0}^{s}Y_{A}(u)\lambda_{A}(u)du+\frac{1}{n}\int_{0}^{s}Y_{B}(u)\lambda_{B}(u)du\\ & =\frac{1}{n}M(s)+\frac{1}{n}\int_{0}^{s}Y_{A}(u)\lambda_{A}(u)du+e^{-n^{-1/2}\gamma }\cdot \frac{1}{n}\int_{0}^{s}Y_{B}(u)\lambda_{A}(u)du\\ & \to 0+\int_{0}^{s}y_{A}(u)\lambda_{A}(u)du+\int_{0}^{s}y_{B}(u)\lambda_{A}(u)du=\int_{0}^{s}y(u)\lambda_{A}(u)du. \end{array}$$

The convergence of the integrals holds using (40), Slutsky theorem and proposition A3 (a). To prove the assertion regarding $\mu_{k}(s)$ , notice that in the limit n→∞ in probability

$$\begin{array}{rl} & n^{-1/2}\int_{0}^{s}L(u)\{\lambda_{A}(u)-\lambda_{B}(u)\}du\\ & =n^{1/2}(1-e^{-n^{-1/2}\gamma })\int_{0}^{s}\frac{Y_{B}(u)/n}{Y_{A}(u)/n+Y_{B}(u)/n}\frac{Y_{A}(u)}{n}\lambda_{A}(u)du\\ & \to \frac{\gamma \cdot v}{(1+v{)}^{2}}\int_{0}^{s}y_{}(u)\lambda_{A}(u)du=\frac{\gamma \cdot v}{(1+v{)}^{2}}\cdot \pi (s). \end{array}$$

The convergence of the integrals holds again using (40), Slutsky theorem and proposition A3 (a). In the last equality we made use of (46). Since $J_{A}(u)\to 1$ and $J_{B}(u)\to 1$ as n→∞ , we likewise conclude

$$\begin{array}{rl} & \lim_{n\to \infty }n^{1/2}\int_{0}^{s}\{J_{A}(u)\lambda_{A}(u)-J_{B}(u)\lambda_{B}(u)\}du\\ & =\lim_{n\to \infty }\{n^{1/2}(1-e^{-n^{-1/2}\gamma })\}\int_{0}^{s}\lambda_{A}(u)du=\gamma \Lambda_{A}(s). \end{array}$$

With similar arguments, we used to show (44), we can conclude

$$\begin{array}{rl} \left\langle M^{LR},M^{\Delta }\rangle (s\right) & =\sum_{x=A,B}\int_{0}^{s}\frac{Y_{-x}(u)/n}{Y_{A}(u)/n+Y_{B}(u)/n}\lambda_{x}(u)du\\ & \to \frac{v}{1+v}\int_{0}^{s}\lambda_{A}(u)du+\frac{1}{1+v}\int_{0}^{s}\lambda_{A}(u)du=\Lambda_{A}(s) \end{array}$$

as well as

(47)
$$\begin{array}{rl} \left\langle M^{LR}\rangle (s\right) & =\sum_{x=A,B}\int_{0}^{s}{\left\{\frac{Y_{-x}(u)/n}{Y_{A}(u)/n+Y_{B}(u)/n}\right\}}^{2}\{Y_{x}(u)/n\}\cdot \lambda_{x}(u)du\\ & \to {\left(\frac{v}{1+v}\right)}^{2}\int_{0}^{s}\frac{y(u)}{1+v}\lambda_{A}(u)du+{\left(\frac{1}{1+v}\right)}^{2}\int_{0}^{s}\frac{v\cdot y(u)}{1+v}\lambda_{A}(u)du\\ & =\frac{v}{(1+v{)}^{2}}\int_{0}^{s}y(u)\lambda_{A}(u)du=\frac{v}{(1+v{)}^{2}}\pi (s). \end{array}$$

In the last equality, we made use of (46).  □

*Proof of equations (13) and (14)*. Our preliminary work enables a quick calculation of $\sigma_{LR}(s)$ and $\sigma_{\Delta }(s)$ . For this purpose we first take a closer look at $y_{A}(u)$ . Under the no loss to follow up and uniform recruitment assumptions, it holds

(48)
$$P(C_{i}>u)=P(C_{i}+E_{i}>u+E_{i})=P(a+f-u>E_{i})=\left\{ \begin{array}{cc} 1 & u\leq f\\ 0 & u\geq a+f\\ \frac{a+f-u}{a} & else. \end{array}\right.$$

Analogue to proof of theorem 1 and with use of (45) it holds

(49)
$$\left\langle M_{n}^{\Delta }\right\rangle (s)\frac{P}{n\to \infty }\frac{(1+v{)}^{2}}{v}\int_{0}^{s}\frac{\lambda_{A}(u)}{S_{A}(u)\cdot P(C_{i}>u)}du$$

Using equation (48), the identity $\lambda_{A}(u)=-S_{A}^{\prime }(u)/S_{A}(u)$ and the substitution $z=S_{A}(u)$ , we get equation (13). With (47) and (48), we also conclude

$$\left\langle M_{n}^{LR}\right\rangle (s)\frac{P}{n\to \infty }\frac{v}{(1+v{)}^{2}}\int_{0}^{s}S_{A}(u)\cdot P(C_{i}>u)\lambda_{A}(u)du$$

Using the same identities and substitution as in (49), we get equation (14).  □

***A seamless phase II/III design***

In this section we elaborate application of our design algorithm in the context of a two–armed randomized seamless phase II/III survival trial. In the phase II part, we assume that the two treatments are compared regarding the short–term endpoint *survival rate at time $s_{0}$ *. I.e. as phase II part, we consider a local level α test of the confirmatory null hypothesis $H_{0,1}\;:\;S_{A}(s_{0})=S_{B}(s_{0})$ on the $s_{0}$ survival rates using the rejection region

(50)
$$R_{1}\;:\;=\{B_{1}>\Phi^{-1}(1-\alpha )\}.$$

$R_{1}$ realizes a single step test of $H_{0,1}$ . Only in the case of rejection of $H_{0,1}$ (i.e. $B_{1}>\Phi^{-1}(1-\alpha )$ ), we continue the trial in order to compare the two treatments also regarding long–term survival. I.e. as phase III part, we consider a local level α test of the confirmatory null hypothesis $H_{0,2}\;:\;S_{A}(s)=S_{B}(s)$ for all $0\leq s\leq s_{\max}$ for some prefixed $s_{\max}>0$ using the rejection region

(51)
$$R_{2}\;:\;=\{Z_{1}^{*}\geq u_{1}\}\cup \{Z_{1}^{*}<u_{1},Z_{2}^{*}\geq u_{2}\}.$$

$R_{2}$ realizes a two-step test of $H_{0,2}$ . It makes sense to synchronize the analysis of $H_{0,1}$ with the interim analysis of $H_{0,2}$ . Notice that, we may choose $u_{1}\;:\;=\infty$ if we wish to refrain from testing $H_{0,2}$ already at the interim analysis and that adjustment to multiple testing is done by hierarchical testing in the order $H_{0,1}$ followed by $H_{0,2}$ , i.e. we reject $H_{0,2}$ to the multiple level α if and only if $H_{0,1}$ and $H_{0,2}$ are both rejected by their local level α tests. $H_{0,1}$ can be rejected to the multiple level α if $H_{0,1}$ is rejected locally.

At the interim analysis, we are free to perform a data–dependent sample size recalculation based on the observed interim log–rank statistic $Z_{1}^{*}$ and the observed difference in the short term response $B_{1}$ .

For a sample size calculation algorithm we have to apply the methodology presented in section ‘Initial sample size calculation’ to the rejection region

(52)
$$R_{3}\;:\;=R_{1}\cap R_{2}=\{B_{1}>\Phi^{-1}(1-\alpha ),Z_{1}^{*}\geq u_{1}\}\cup \{B_{1}>\Phi^{-1}(1-\alpha ),Z_{1}^{*}<u_{1},Z_{2}^{*}\geq u_{2}\}$$

with power defined by the probability $P_{K}(R_{3})$ under some planing alternative K .
